# Rabies in the Endemic Region of Algeria: Knowledge, Attitude and Practice (KAP) Survey among University Students

**DOI:** 10.3390/ani14152193

**Published:** 2024-07-27

**Authors:** Mohamed Lounis, Mousab Zarif, Zoubida Zeroug, Salma Soulaf Ferial Brahimi, Zineb Meddour

**Affiliations:** 1 Laboratoire d’Exploration et Valorisation des Écosystèmes Steppiques, Faculty of Natural and Life Sciences, University of Djelfa, P.O. Box 3117, Djelfa 17000, Algeria; 2 Department of Biology, Faculty of Natural and Life Sciences, University of Djelfa, P.O. Box 3117, Djelfa 17000, Algeria; zarifmoussab@gmail.com (M.Z.); zoubidazerouk@gmail.com (Z.Z.); soulef.selma.feriel@gmail.com (S.S.F.B.); zinebmddr15@gmail.com (Z.M.)

**Keywords:** rabies, knowledge, attitude, practice, KAP, students, Algeria

## Abstract

**Simple Summary:**

This cross-sectional study was conducted to evaluate the knowledge, practices, and attitudes of university students in the endemic region of Algeria in regard to rabies in the remaining years before the plan to eliminate dog-mediated rabies concludes in 2030. This disease is still spreading in Algeria, killing around 15 humans and more than 380 dogs per year despite the panel of applied measures that have been put in place over multiple years. Overall, a medium level of knowledge was obtained among the 409 surveyed students, with low levels reported among certain categories, including students studying in other fields than medical sciences, and there were some gaps of knowledge in terms of disease transmission, symptoms and risk factors. Regarding their attitudes, if an overall positive attitude was reported, some negative attitudes were also observed, especially the belief in the necessity of killing dogs who bite someone regardless of their vaccine status (59.2%). The importance of bite-wound washing is still ignored by most students. These results suggest the need to increase the level of knowledge of the Algerian students, and the public in general, in regard to this deadly disease through awareness campaigns.

**Abstract:**

(1) Background: Rabies is a neglected zoonosis considered to be one of the most significant health threats in the world, responsible of thousands of deaths globally. Algeria is one of the main countries of concern, with more than 15 deaths and more than 100,000 bites by rabid-suspected animals being reported annually. This study was conducted to evaluate the level of knowledge, attitudes and practice (KAP) toward this disease among university students in the endemic region of Algeria. (2) Methods: a cross-sectional online survey was conducted between June 2023 and March 2024 using a self administered questionnaire. (3) Results: a total of 409 students completed the questionnaire. Overall, 91.2% of them were singles, 70.7% were females, and two-thirds (66%) were between their twenties and thirties in regard to age. The majority were studying for Bachelor’s degrees, and the most represented fields of study were Humanities (31.5%) and Natural and Life Sciences (29.1%).The findings revealed a moderate level of knowledge (63% of correct responses), a positive attitude, and appropriate practices. However, the surveyed students have certain gaps in their information regarding disease transmission, its risk factors, and its clinical signs. In addition, some of them have also shown some negative attitudes, including approving of the systematic killing of a dog who bites someone regardless of its vaccine status (59.2%), and inappropriate practices, especially in regard to unawareness of the importance of early washing of bite wounds, which was reported among 64.5% of the surveyed students. The study also revealed the association of health sciences education with knowledge (AOR: 2.723, CI 95%: 1.317–5.634), attitude (AOR: 2.306, CI 95%: 1.113–4.779) and practice (AOR: 3.560, CI 95%: 1.632–7.769), and the effect of the high level of knowledge on the attitude of surveyed students (AOR: 1.607, CI 95%: 1.052–2.456). (4) Conclusion: These results provide the first report regarding rabies KAP among Algerian university students. Based on these results, the health deciders could adopt their preventive strategy by raising awareness of the less-informed categories, which would consequently affect their behaviors regarding this fatal disease.

## 1. Introduction

Despite being known of since the Mesopotamian era, about 3000 BCE, and despite the significant progress in novel medical technologies and the implementation of various control programs, rabies continues to pose a serious threat to human and animal health, remaining one of the most significant veterinary and public health concerns in the 21st century [1,2,3]. Based on global estimates, there are more than 59,000 rabies-related deaths annually in 150 countries across all continents (excluding Antarctica). Most of these cases, about 95%, occur in African and Asian countries [4], with 99% of cases being dog-mediated through bite exposures [1,5].

This disease, caused by a single-stranded RNA neurotropic virus of the family Rhabdoviridae and the genus Lyssavirus, is one of the most fatal worldwide, as almost all individuals who develop symptoms of rabies succumb to an acute progressive encephalitis [6,7]. However, mortality can be prevented through prompt and early post-exposure prophylaxis (PEP), which includes wound washing with water and soap, administration of rabies immunoglobulin, and vaccines following animal bites [8,9].

It is evident that mass vaccination of domestic dog populations is the key strategy to preventing human rabies [9,10,11]. This strategy helped to eliminate dog rabies and reduce human cases in multiple countries in Europe and in North and Latin America [6]. These results were attained thanks to one health approach that involves the collaboration of professionals in human, animal, and environmental health [6].

Based on these experiences, a collaborative plan was established by the World Health Organization (WHO), the Food and Agriculture Organization (FAO), the World Organization for Animal Health (WOAH), and the Global Alliance for Rabies Control (GARC) to achieve “zero human deaths from dog-mediated rabies by 2030” [4].

Algeria has also adopted this strategy. In fact, a legislative panel was put in place to ensure mass vaccination of dog populations and the fight against free-roaming dogs [12]. However, despite these measures, this neglected zoonosis is still spreading, resulting in approximately 15 deaths annually. In addition, approximately 120,000 animal bites are recorded every year, with dogs being the primary culprits [13]. In 2019, 382 rabies cases were reported among dogs in the country [14]. Interestingly, all these cases were concentrated in the northern region, with no reported cases (neither in animals nor humans) in the Sahara, located in the southern region of Algeria [12]. These data highlight the necessity of reassessing the rabies prevention strategy in Algeria. One crucial aspect that seems to be lacking is public education about this disease. Enhancing the level of knowledge is likely to positively impact attitudes and practices, ultimately aiding in the fight against rabies [15]. Similar findings have been reported in various public health studies focusing on knowledge, attitudes, and practices (KAP) in endemic countries [8,9,10,15,16,17,18,19,20,21,22,23,24,25,26]. However, there is limited information available on this topic in Algeria. Therefore, the present study was conducted to assess the KAP of Algerian students regarding this disease.

## 2. Materials and Methods

### 2.1. Study Design and Study Area

**Figure 1:**
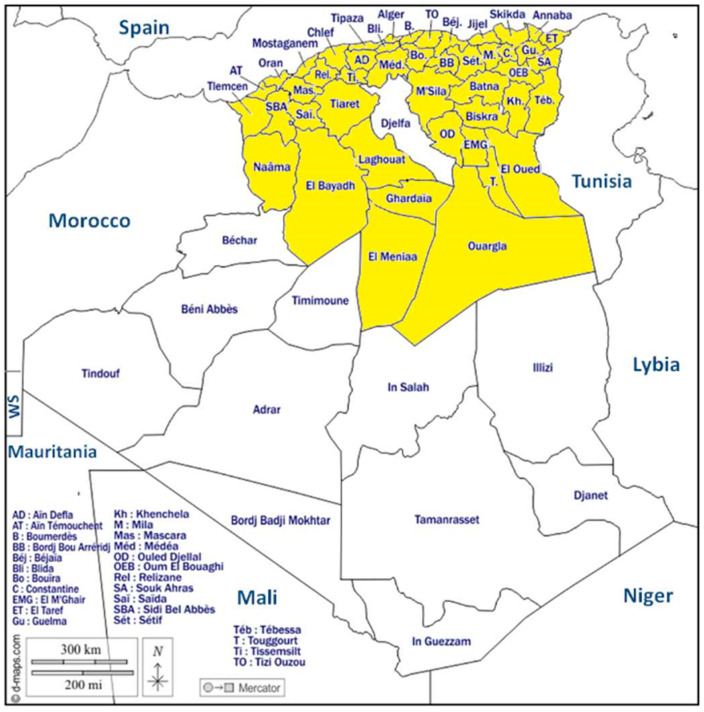
Distribution of the study participants according to the departments of Algeria (Yellow color indicate the departments included in the survey).

This cross-sectional KAP survey targeted students from the endemic departments in Algeria. It was conducted using an online questionnaire with a Google forms link that was shared through social media platforms. The survey was distributed through Facebook/Messenger, WhatsApp groups, or by direct contact. All students aged 18 years and older who were living in departments where human rabies was detected were eligible to participate. Of note, rabies is predominantly reported in the northern and Steppic regions, with some cases also being documented in the northern departments of the Sahara, while the southern (Saharian) region appears to be free from the disease (Figure 1). As a result, students from this region, including the 12 departments listed in Table S1 of the Supplementary Materials, were excluded from the study.

The online survey was conducted in accordance with international standards, including the Declaration of Helsinki and the STROBE guidelines outlined by von Elm et al. in 2007 [27]. Participation was voluntary, and e-consent was obtained before respondents completed the questionnaire. A mandatory question, “Do you agree to participate in this survey?“ was asked. Participants were informed that participation was optional and that they could exit the survey without submitting responses if they so chose. Additionally, they were assured that participation did not entail any financial compensation and that their data would be treated anonymously. The study received approval from the Scientific Committee of the Faculty of Natural and Life Sciences at the University of Djelfa on 25 January 2023 (reference: 29/25 January 2023).

### 2.2. Sample Size

The minimum number of students to include in the sample was calculated using the survey monkey sample size calculator [28] with the following assumptions: an expected proportion of 0.5, a student population of 1,500,000 for the academic year 2023/2024, a confidence level of 95%, and a margin of error of 5%. The calculated number of students needed was 385.

### 2.3. Study Tool

The rabies KAP questionnaire used in this study was extracted from previous studies focusing on this subject [17,18,20,21,22,23,24]. It was disseminated in both the Arabic and French languages. Following an introductory paragraph explaining the study’s objective and the targeted population, and obtaining consent from the students, the questionnaire addressed various aspects. The initial section focused on the demographic and academic characteristics of the participants (age, sex, marital status, education level, field of study, financial status, residential area). It also included inquiries about their involvement with livestock and dog ownership, as well as any history of dog bites. Additionally, students were questioned about the sources they used to gather information about rabies.

The following section was designed to gather information to evaluate the level of knowledge (16 items), attitudes (8 items), and practices (8 items). Students had the opportunity to respond to these items with “yes”, “no”, or “I don’t know”.

To calculate the level of knowledge, attitude, and practice, a correct response was awarded 1 point, while an incorrect response (including responses of “I don’t know”) scored 0 points. The mean scores for knowledge, attitude, and practice were calculated and used to categorize students as having either a high or low level of knowledge, a positive or negative attitude, and good or bad practice based on their scores. Scores above the mean were classified as high knowledge, positive attitude, and good practice, while scores below were considered to be the opposite.

### 2.4. Statistical Analysis

The Excel file sheet of the responses was moved to SPSS version 22 software for Windows, where all statistical analysis were performed assuming a *p* value of 0.05 for significant results. Frequency and scores of knowledge, attitude, and practice were calculated and expressed as percentages, means, and medians, respectively. After categorization of the levels of KAP, associated factors were sought using either Chi squared tests, Fisher tests, or crude Odds Ratios (CORs). Factors that showed a *p* ≤ 0.2 [26] in the univariate analysis were included in the logistic regression model to confirm or infirm the results through the calculation of the adjusted Odd Ratio (AOR).

## 3. Results

### 3.1. Socio-Demographic and Scholar Details

Four hundred and eighteen (418) students replied to the questionnaire of this survey. This number has been reduced to 409 questionnaires following the removal of incomplete responses. The respondents were mostly singles (91.2%), females (70.7%), and aged between 20 and 29 years (66%). The most represented specialties were Humanities (31.5%) and Natural and Life Sciences (29.1%), with Bachelor’s degree students making up the majority (57.9%). The vast majority (85.3%) lived in urban zones, and more than three-quarters (75.1%) had a medium financial level. Regarding animal ownership, 42.8% declared having a relation with livestock breeding, while less than one-fifth (19.6%) had a relation with dog-raising.

Finally, 38.9% declared knowing someone who was bitten by a dog and 8.1% had been bitten (Table 1).

### 3.2. Rabies Knowledge

Results of the study showed that a medium proportion of 63% of correct responses was obtained. The corresponding score was 10.1 ± 3.1 points from a scale of 16 points. Most of the respondents were aware of the existence of rabies in Algeria (83.1%) and the importance of dogs as a potential source of this disease (82.6%). Additionally, a high proportion of the asked students were knowledgeable about some aspects of this disease, including the role of animals (79.2%) or dog bites (73.8%) in the transmission of the disease and its deadly nature (72.9%). However, some aspects showed low levels of correct responses. The lowest rates were obtained for items related to the age of dogs’ vaccination and the possibility of transmission of rabies between humans, with 24.9% and 34.5% of correct responses, respectively (Table 2).

**Figure 2:**
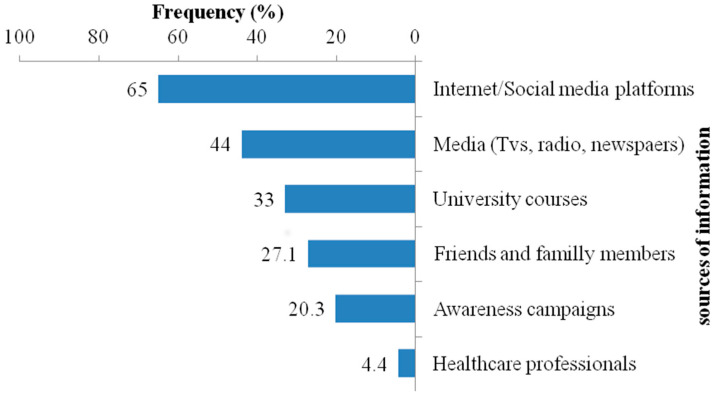
Source of information about rabies.

The sources of knowledge regarding this disease among the respondents included Internet surfing and social networking (65%), traditional media such as television, radio, and newspapers (44%), university and/or school courses (33%), and additional sources such as family/friends discussions, awareness campaigns, and healthcare professionals (Figure 2).

### 3.3. Rabies-Related Attitude

The results revealed that the respondents generally adopted a positive attitude toward this disease. Most of the respondents reported that they would go to the hospital if bitten by a dog (96.6%), believe in the importance of dog vaccination (93.6%), and acknowledge the danger of playing with unknown dogs (92.4%). Lower rates were also reported regarding attitudes toward children playing with dogs (81.9% were opposed), allowing unknown dogs to roam around them (70.9%), or allowing dogs to wander to obtain food (65.5%).

Finally, 59.2% of the respondents believed that a dog that bites someone should be caught and killed, while 26.9% were opposed to confining dogs (Table 3). The respondents scored an average of 5.9 ± 1.3 out of a total of 8 points.

### 3.4. Rabies-Related Practice

Out of the 409 respondents, 81.2% said that traditional medicines should not be used for dog bites, while only 35.5% said that washing dog bite wounds with soap constitutes the first step in post-exposure rabies treatment. Additionally, 24.2% of the participants were against the castration of dogs to prevent the disease, while 44.3% were in favor of this practice (Table 4).

### 3.5. Factors Associated with the Level of Knowledge, Attitude, and Practice

The findings of the univariate analysis revealed that, except for marital status (*p* = 0.495), living area (*p* = 0.906), livestock raising (*p* = 0.648), and dog bite status (*p* = 0.289), all other factors were significantly associated with the level of knowledge.

For attitudes, individuals over 30 years old (*p* = 0.002), those residing in rural areas (*p* = 0.02), those involved in livestock raising (*p* = 0.007), and those having a high level of knowledge (*p* = 0.004) were significantly associated with a positive attitude.

Finally, the factors associated with good practice included education in medical sciences (*p* = 0.001), involvement in dog raising (*p* = 0.023), acquaintance with someone who was bitten by a dog (*p* = 0.005), and possessing a high level of knowledge (*p* = 0.02) (Table 5).

By applying logistic regression, the model demonstrated that females were associated with a low level of knowledge (AOR: 0.533, CI 95%: 0.336–0.845), while students in Natural and Life Sciences (AOR: 1.95, CI 95%: 1.205–3.155) and medical sciences (AOR: 2.723, CI 95%: 1.317–5.634) were predictors of high knowledge. The latter category was also linked to a positive attitude (AOR: 2.306, CI 95%: 1.113–4.779) and good practice (AOR: 3.560, CI 95%: 1.632–7.769).

The other factors associated with a positive attitude included residing in rural areas (AOR: 1.842, CI 95%: 1.017–3.334), being involved in livestock breeding (AOR: 1.591, CI 95%: 1.040–2.434), and possessing a high level of knowledge (AOR: 1.607, CI 95%: 1.052–2.456), while the determinants of good practice comprised students who were acquainted with someone who had experienced dog bites (AOR: 1.545, CI 95%: 1.000–2.386) (Table 6).

## 4. Discussion

The study evaluated rabies-related knowledge, attitudes, and practices among university students in the endemic region of Algeria, where rabies continues to persist and cause fatalities [3,29] despite being recognized as a major disease since 1984 and having legal measures in place in regard to its prevention since 1996 [12,30]. This area of study has often been overlooked, with no prior research having been conducted in the country. Therefore, the findings of this study are anticipated to offer fundamental insights into students’ knowledge in the endemic region which can be utilized in awareness initiatives to combat this lethal disease.

Overall, a medium level (63%) of knowledge was obtained in this study. This level appears to be higher than the knowledge levels reported among various communities in certain African and South Asian countries [16,26,31,32,33,34]. Higher rates were also documented among other cohorts such as dog owners in Ghana [19], scholar students in Thailand [35], and the population of the Amhara region in Ethiopia [20]. Nevertheless, these results should be interpreted with caution considering the variations in the items and scales utilized.

Looking in more detail, if the respondents were highly aware of the endemicity of the disease in Algeria, its deadly nature, and the role of dogs as the main source for human cases, they were less knowledgeable regarding the other methods of its transmission, its clinical signs, and its prevention. The link between dogs and rabies is a common finding in almost all previous studies [20,24,33]. This result is not surprising, knowing that 99% of human cases originated from a dog bite according to the World Health Organization (WHO) [4]. The same observation was also reported in Algeria [12]. Note that the name of rabies in the Arabic language (*Alkalab* الكَلَبْ) is derived from dog (*Alkalb* الكَلْبْ), which could explain the link between dogs and rabies among the respondents. However, the importance of other animals was overlooked in our study. A similar observation was also previously reported in Morocco [24] and in Nepal [21]. These findings suggest the need to sensitize the population about the risk that other animals could pose in the persistence of rabies, especially when considering that this disease is also common among cattle (bovine, ovine/caprine, equine), and cases were also reported among wild animals in Algeria [12,14].

Another missing aspect of the respondents regarding rabies is related to clinical signs. Indeed, while 72.9% of the respondents were aware that rabies could be transmitted by saliva, a considerable portion of 34% and 39.4% were unaware that a sudden change in attitude, excessive salivation, and the tendency to bite anything are a potential signs of rabies, respectively. In addition, 55.3% of them only knew that the disease is associated with nervous signs. These rates are far lower than the results previously reported in different African (Rwanda, Ethiopia, Senegal) and Asian (Nepal) countries [8,15,20,33,36]. Contrarily, Awuni et al. [19] reported a low level of knowledge of clinical signs in humans among dog owners in Ghana, while excessive salivation was known as a sign of rabies by only 38% of school children of South Bhutan in Thailand [35] and 37% of the younger population in Pakistan [16]. These results showed the fragmented information of the participants regarding clinical signs of rabies, suggesting the need to raise the level of knowledge by focusing on the items with the lowest level of correct responses.

Contrary to findings in Ethiopia and Tanzania, where almost all household heads obtained information about rabies from informal sources [23,26], students in our study primarily mentioned the Internet/social media and traditional media as their sources of information. This variance could be attributed to age differences, as younger individuals are typically more engaged with social networking platforms compared to older individuals. However, a significant concern arises from the limited involvement of healthcare providers and awareness campaigns in disseminating information about the disease in our study. This result underscores the necessity of intensifying efforts to inform and educate the population in order to eradicate this disease.

The significant finding regarding attitude is that 59.2% of respondents believed that a dog that bites someone should be caught and killed regardless of its vaccination status. Similar views have been reported in various countries such as Rwanda [8], Ethiopia [20,23], Morocco [24], Nepal [21], and Pakistan [16,37]. It is important to note that the appropriate measure in such situations is to place the dog in quarantine for 10 days to monitor any potential changes that could indicate or rule out rabies infection [38].

Regarding students’ practices, it is apparent that only 35.5% were in favor of washing the wound with soap after a dog bite. While this practice was reported by 86% of school children and 60% among the rural community in Bangladesh, it was overlooked by the majority of the population in different countries. For instance, rates of 3.2% in Pakistan [16], 8–30.7% in Ethiopia [20,23,36], 20% in Zimbabwe [34], and 30% in Uganda [31] have been cited. More importantly, Tiwari et al. [9], found that individuals with higher knowledge about rabies control practices were oppositely unaware of the importance of wound washing.

Of note, immediate wound washing with soap and water for a minimum of 15 min is the first step recommended by the World Health Organization (WHO) and national health authorities [39] in post-exposure prophylaxis. This practice is associated with a 50% increase in the survival of affected animals and can reduce the development of rabies by one-third [40]. Therefore, improving awareness about the importance of this basic and simplistic method could significantly reduce the risk of developing the disease. The subsequent essential step is the timely administration of rabies immunoglobulin and vaccines, both of which are available in Algeria (where two types of vaccines are available: a cell culture vaccine for immune-compromised individuals and a tissue vaccine produced from mice brains by the Pasteur Institute of Algeria, which is the most commonly used one) [41].

The positive aspect of this study is that only a small percentage of the surveyed students indicated a preference for traditional healers in the event of a dog bite. Despite the lack of scientific evidence supporting their effectiveness, the use of these remedies is widespread in various countries [16,20,21,23,36,37]. Considering that this practice is accountable for a significant number of fatal rabies cases, increasing awareness about the risks associated with traditional healers is crucial for safeguarding people’s lives.

In this study, Medical (AOR: 2.723, CI 95%: 1.317–5.634) and Natural and Life (AOR:1.95, CI 95%: 1.205–3.155) Sciences education and male sex (AOR: 0.533, CI 95%: 0.336–0.845) were the only determinants that increased the level of knowledge. While the role of the field of study is understandable, as this subject may be included in the curriculum of medical and life sciences programs, the impact of sex requires further investigation. Although similar findings were reported by Bahiru et al. [20] and Bihon et al. [23] in Ethiopia and Sambo et al. [17] in Tanzania, most studies have not identified a significant difference between males and females [8,19,34,37]. One possible explanation could be that females are less likely to experience dog bites [41], leading them to place less importance on understanding this disease.

Furthermore, students in the medical sciences exhibited a higher likelihood of having a positive attitude (AOR: 2.306, CI 95%: 1.113–4.779) and good practice (AOR: 3.560, CI 95%: 1.632–7.769). These findings are significant given the crucial role these students play as future healthcare providers and sources of public information. In contrast, there should be increased focus on students from other disciplines, considering them a priority group in awareness initiatives aimed at enhancing rabies knowledge.

In contrast, students who had a history of dog bites and those involved in dog raising were surprisingly not associated with any of the levels of knowledge, attitude, and practice. For example, the positive impact of pet ownership on knowledge levels is a consistent finding in previous studies [17,20,26,34,37]. A similar trend was also noted for attitude [10] and practice [17,21] regarding this disease.

Regarding the history of dog bites, while the same result was shared by Dhakal et al. [22], Bihon et al. [23], and Hagos et al. [26], Subedi et al. [33] found that, even though a history of family exposure to dog bites was not associated with the level of knowledge, it could positively affect attitudes and practices in regard to rabies. Surprisingly, a study from China revealed that exposure to animals two or more times was associated with negative practices regarding rabies control [18].

Another intriguing finding is the correlation between rural students (AOR: 1.842, CI 95%: 1.017–3.334), individuals involved in livestock breeding (AOR: 1.591, CI 95%: 1.040–2.434), and those exhibiting a high level of knowledge (AOR: 1.607, CI 95%: 1.052–2.456) with a positive attitude. Previous research has already established a link between rabies knowledge, attitude, and rural residency [23], although conflicting results have also been reported [20].

Taken together, while the results of Hagos et al. [26] were surprisingly in favor of the association between knowledge, attitude, and practice, other researchers have also confirmed that the level of knowledge positively influences attitude, which in turn affects practice regarding rabies [15,25,32]. These findings underscore the significance of educational programs aimed at enhancing knowledge levels, attitudes, and practices, which can be instrumental in the prevention strategy against this disease.

As with any KAP study, our study has its strengths and limitations. The most significant strength is related to the timing and exclusivity of this study. It is the first of its kind in Algeria to address rabies KAP. Additionally, it focused on university students, who are key in disseminating information to the general population. Moreover, this study was conducted just a few years before the WHO’s deadline to eliminate rabies by 2030. Therefore, its findings could assist in assessing preventive strategies and implementing any needed adjustments.

Regarding the limitations, the sampling method represents the most significant one. In fact, this study is conducted among students and cannot be generalized to the general population. It may also marginalize the most susceptible populations (habitant of rural communities, dog owners) that could provide more pertinent and detailed information regarding this disease. In addition, conducted through an online questionnaire, this study may have marginalized certain categories, particularly students with limited Internet access, especially those residing in rural areas. Another limitation is the influence of social desirability in this type of study. Respondents might have sought information online before answering to provide what they perceived as the ‘correct’ response. Additionally, they may have been inclined to report positive attitudes and good practices, a factor that is challenging to control in such studies.

## 5. Conclusions

In conclusion, despite the aforementioned limitations, this study provides valuable insights into the level of KAP among Algerian university students. Overall, a moderate level of knowledge was observed, with students demonstrating gaps in understanding certain aspects of the disease. However, they generally exhibited a positive attitude and good practices. The study also identified some associated factors, with medical students being notably linked to a high level of KAP and a positive attitude correlating with a high level of knowledge.

To complement these findings, conducting additional surveys targeting the most vulnerable groups and those unable to participate in online surveys, particularly the rural population, could offer a more comprehensive understanding of rabies KAP in Algeria. This enhanced insight would aid in the prevention of this deadly disease.

## Figures and Tables

**Table 1:** Students’ socio-demographic and educational features.

Variables	Outcomes	Number	Frequency (%)
Age	18–19 years	95	23.2
	20–29 years	270	66.0
	30–39 years	29	7.1
	Over 40 years	15	3.7
Sex	Female	289	70.7
	Male	120	29.3
Marital status	Married	36	8.8
	Single	373	91.2
Educational level	Bachelor’s Degree	237	57.9
	Master’s Degree	150	36.7
	Postgraduates	22	5.4
Faculty	Economics	55	13.4
	Humanities	129	31.5
	Medical Sciences	44	10.8
	Natural and Life Sciences	119	29.1
	Sciences and Technology	62	15.2
Residence	Rural	61	14.9
	Urban	348	85.1
Family standard of living	High	70	17.1
	Low	31	7.6
	Medium	308	75.3
Livestock breeding	Yes	175	42.8
	No	234	57.2
Dog ownership	Yes	80	19.6
	No	329	80.4
Knowing one with dog bites	Yes	159	38.9
	No	250	61.1
Bitten by a dog	Yes	33	8.1
	No	376	91.9

**Table 2:** Rabies-related knowledge of the study participants.

Items	Yes		No		I Don’t Know	
	Number	%	Number	%	Number	%
Rabies exists in Algeria	340	83.1	6	1.5	63	15.4
Rabies is a deadly disease	298	72.9	36	8.8	75	18.3
Infectious agent of rabies infects nerves	226	55.3	8	2.0	175	42.8
All animals could be infected and transmit rabies	216	52.8	77	18.8	116	28.4
Dogs are the possible source of rabies in Algeria	338	82.6	21	5.1	50	12.2
All categories of humans could be affected by rabies	222	54.3	101	24.7	86	21.0
Animal bites could transmit the infectious agent to a healthy animal	324	79.2	13	3.2	72	17.6
Rabies is transmitted by saliva	298	72.9	19	4.6	92	22.5
Transmission from human to human by contact is possible	141	34.5	93	22.7	175	42.8
Dog bites increase the risk of getting rabies	302	73.8	33	8.1	74	18.1
A docile dog that suddenly turns aggressive may have rabies	270	66.0	50	12.2	89	21.8
Excessive foamy salivation and the tendency to bite anything are signs of rabies in dogs	248	60.6	27	6.6	134	32.8
It is against the law not to vaccinate dogs against rabies	244	59.7	39	9.5	126	30.8
Age of vaccination of dogs is 3 months	102	24.9	23	5.6	284	69.4
Vaccination of dogs against rabies must be repeated every year	262	64.1	27	6.6	120	29.3
Registering dogs could help to control rabies	293	71.6	14	3.4	102	24.9
Average	-	63.0	-	9.0	-	28.0
Mean Score	10.1 ± 3.1					
Median	10.0					

**Table 3:** Rabies-related attitude of the study participants.

Items	Yes		No		I Don’t Know	
	Number	%	Number	%	Number	%
I do not allow stray dogs to roam freely around me	290	**70.9**	97	23.7	22	5.4
A dog that bites someone should be caught and killed	242	59.2	133	**32.5**	34	8.3
If I am bitten by a dog, I will go to the hospital	395	**96.6**	7	1.7	7	1.7
It is good to let dogs wander to obtain food because it makes them grow quickly	67	16.4	268	**65.5**	74	18.1
It is inhumane/bad to confine your dog(s)	110	**26.9**	245	**59.9**	54	13.2
It is good not to play with unknown dogs	378	**92.4**	23	5.6	8	2.0
Keeping dogs unvaccinated against rabies is dangerous and should be avoided	383	**93.6**	14	3.4	12	2.9
Children should be allowed to play with dogs	43	10.5	335	**81.9**	31	7.6

Bold character indicates the positive attitude.

**Table 4:** Rabies-related practice of the study participants.

Items		Yes		No		I Don’t Know	
		Number	%	Number	%	Number	%
Is it good to vaccinate your dog(s)		385	**94.1**	13	3.2	11	2.7
Is it good to wash dog bites with soap		145	**35.5**	80	19.6	184	45.0
It is good to have a cage for your dog(s)		310	**75.8**	32	7.8	67	16.4
It is not a good practice to castrate/neuter dogs		99	24.2	181	44.3	129	31.5
If a person is bitten by a dog, what should be done?	Take the victim to a pharmacy for treatment	143	35.0	247	60.4	19	4.6
	Treat with traditional medicine	34	8.3	332	81.2	43	10.5
	Take the victim to a veterinary clinic	64	15.6	312	76.3	33	8.1
	Take to the hospital	390	**95.4**	10	2.4	9	2.2
	Nothing	21	5.1	357	87.3	31	7.6

Bold character indicates the good practice.

**Table 5:** Univariate analysis of the factors associated with rabies KAP.

		Knowledge			Attitude			Practice		
		High	Low	*p* Value	Positive	Negative	*p* Value	Good	Poor	*p* Value
		*n* (%)	*n* (%)		*n* (%)	*n* (%)		*n* (%)	*n* (%)	
Age category	18–19 yo	41 (43.2)	54 (56.8)	0.121	42 (44.2)	53 (55.8)	0.920	50 (52.6)	45 (47.4)	0.945
	20–29 yo	134 (49.6)	136 (50.4)	**0.002**	108 (40.0)	162 (60.0)	**0.032**	136 (50.4)	134 (49.6)	0.271
	Over 30 yo	30 (68.2)	14 (31.8)	**0.011**	29 (65.9)	15 (34.1)	**0.002**	28 (63.6)	16 (36.4)	0.112
Sex	Female	134 (46.4)	155 (53.6)	**0.018**	126 (43.6)	163 (56.4)	0.916	152 (52.6)	137 (47.4)	0.864
	Male	71 (59.2)	49 (40.8)		53 (44.2)	67 (55.8)		62 (51.7)	58 (48.3)	
Marital status	Married	20 (55.6)	16 (44.4)	0.495	21 (58.3)	15 (41.7)	0.065	20 (55.6)	16 (44.4)	0.684
	Single	185 (49.6)	188 (50.4)		158 (42.4)	215 (57.6)		194 (52.0)	179 (48.0)	
Educational level	Bachelor’s Degree	103 (43.5)	134 (56.5)	**0.002**	95 (40.1)	142 (59.9)	0.078	115 (48.5)	122 (51.5)	0.071
	Master’s Degree	88 (58.7)	62 (41.3)	**0.009**	75 (50.0)	75 (50.0)	0.053	83 (55.3)	67 (44.7)	0.354
	PG	14 (63.6)	8 (36.4)	0.273	9 (40.9)	13 (59.1)	0.829	16 (72.7)	6 (27.3)	0.077
Faculty	MS	27 (61.4)	17 (38.6)	0.114	25 (56.8)	19 (43.2)	0.065	33 (75.0)	11 (25.0)	**0.001**
	NLS	68 (57.1)	51 (42.9)	**0.069**	45 (37.8)	74 (62.2)	0.120	68 (57.1)	51 (42.9)	0.211
	Other	110 (44.7)	136 (55.3)	**0.007**	109 (44.3)	137 (55.7)	0.785	113 (45.9)	133 (54.1)	**0.001**
Residence	Rural	31 (50.8)	30 (49.2)	0.906	35 (57.4)	26 (42.6)	**0.020**	36 (59.0)	25 (41.0)	0.256
	Urban	174 (50.0)	174 (50.0)		144 (41.4)	204 (58.6)		178 (51.1)	170 (48.9)	
Family standard of living	High	37 (52.9)	33 (47.1)	0.615	32 (45.7)	38 (54.3)	0.718	35 (50.0)	35 (50.0)	0.669
	Low	21 (67.7)	10 (32.3)	**0.041**	15 (48.4)	16 (51.6)	0.589	16 (51.6)	15 (48.4)	0.934
	Medium	147 (47.7)	161 (52.3)	0.091	132 (42.9)	176 (57.1)	0.518	163 (52.9)	145 (47.1)	0.672
Livestock breeding	Yes	90 (51.4)	85 (48.6)	0.648	90 (51.4)	85 (48.6)	**0.007**	98 (56.0)	77 (44.0)	0.198
	No	115 (49.1)	119 (50.9)		89 (38.0)	145 (62.0)		116 (49.6)	118 (50.4)	
Dog ownership	Yes	48 (60.0)	32 (40.0)	**0.049**	33 (41.3)	47 (58.8)	0.613	51 (63.8)	29 (36.3)	**0.023**
	No	157 (47.7)	172 (52.3)		146 (44.4)	183 (55.6)		163 (49.5)	166 (50.5)	
Knowing one with dog bites	Yes	93 (58.5)	66 (41.5)	**0.007**	78 (49.1)	81 (50.9)	0.085	97 (61.0)	62 (39.0)	**0.005**
	No	112 (44.8)	138 (55.2)		101 (40.4)	149 (59.6)		117 (46.8)	133 (53.2)	
Bitten by a dog	Yes	20 (58.8)	14 (41.2)	0.289	19 (55.9)	15 (44.1)	0.137	22 (64.7)	12 (35.3)	0.131
	No	185 (49.3)	190 (50.7)		160 (42.7)	215 (57.3)		192 (51.2)	183 (48.8)	
Knowledge score	High	.	.	.	104 (50.7)	101 (49.3)	**0.004**	119 (58.0)	86 (42.0)	**0.020**
	Low	.	.		75 (36.8)	129 (63.2)		95 (46.6)	109 (53.4)	

PG: Postgraduates; MS: Medical Sciences; NLS: Natural and Life Sciences. Significant results at *p* ≤ 0.05 are indicated in bold character; yo: years old.

**Table 6:** Logistic regression of rabies-related KAP determinants.

		Knowledge		Attitude		Practice	
		AOR (CI 95%)	*p* Value	AOR (CI 95%)	*p* Value	AOR (CI 95%)	*p* Value
Age	18–19 yo	0.478 (0.194–1.178)	0.109	0.594 (0.219–1.611)	0.306	0.843 (0.350–2.035)	0.705
	20–29 yo	0.483 (0.231–1.012)	0.054	0.426 (0.179–1.011)	0.053	0.654 (0.319–1.340)	0.246
	>30 yo	Ref.		Ref.		Ref.	
Sex	Female	0.533 (0.336–0.845)	**0.007**	-		-	
	Male	Ref.					
Marital status	Married	-		1.052 (0.418–2.649)	0.914	-	
	Single			Ref.			
Educational level	Bachelor’s Degree	0.742 (0.265–2.08)	0.57	1.541 (0.546–4.351)	0.415	0.491 (0.167–1.445)	0.196
	Master’s Degree	1.342 (0.477–3.774)	0.577	2.363 (0.850–6.570)	0.099	0.662 (0.227–1.934)	0.451
	PG	Ref.		Ref		Ref.	
Faculty	MS	2.723 (1.317–5.634)	**0.007**	2.306 (1.113–4.779)	**0.025**	3.560 (1.632–7.769)	**0.001**
	NLS	1.95 (1.205–3.155)	**0.007**	0.752 (0.462–1.223)	0.251	1.508 (0.945–2.407)	0.085
	Other	Ref.		Ref.		Ref.	
Residence	Rural	-		1.842 (1.017–3.334)	**0.044**	-	
	Urban			Ref.			
Family standard of living	High	1.28 (0.732–2.239)	0.387				
	Low	2.257 (0.98–5.202)	0.056	-		-	
	Medium	Ref.					
Livestock breeding	Yes	-		1.591 (1.040–2.434)	**0.032**	1.114 (0.726–1.710)	0.621
	No			Ref.		Ref.	
Dog ownership	Yes	1.489 (0.879–2.52)	0.139	-		1.614 (0.921–2.829)	0.095
	No	Ref.				Ref.	
Knowing one with dog bites	Yes	1.394 (0.902–2.155)	0.135	1.141 (0.733–1.775)	0.559	1.545 (1.000–2.386)	**0.050**
	No	Ref.		Ref.		Ref.	
Bitten by a dog	Yes	-		1.233 (0.578–2.631)	0.588	1.395 (0.633–3.070)	0.409
	No			Ref.		Ref.	
Knowledge score	High	-		1.607 (1.052–2.456)	**0.028**	1.245 (0.820–1.891)	0.304
	Low			Ref.		Ref.	

PG: postgraduates; MS: medical sciences; NLS: Natural and Life Sciences. Significant results at *p* ≤ 0.05 are indicated in bold characters; yo: years old.

## Data Availability

The data that support all findings of this study are available on request from the corresponding author.

## Supplementary Materials

The following supporting information can be downloaded at https://www.mdpi.com/article/10.3390/ani14152193/s1, Table S1: Repartition of the number of participants according to the different departments in Algeria. Table S2: Crude OR of the factors associated with knowledge, attitude and practice about rabies. Annex S1: Questionnaire about Knowledge, Attitudes, and Practices (KAP) about rabies among University Students in the endemic region in Algeria. Annex S2: STROBE Statement—checklist of items that should be included in reports of observational studies.
