# Supplementary material for: Rabies in the Endemic Region of Algeria: Knowledge, Attitude and Practice (KAP) Survey among University Students
Source: Animals (Basel). 2024 Jul 27;14(15):2193. doi: 10.3390/ani14152193 (PMC11311033; doi:10.3390/ani14152193)
Supplement: Supplementary file 1 [file animals-14-02193-s001.zip › animals-3040082-supplementary.pdf]

Table S1: Repartition of the number of participants according to the different departments in Algeria

Department	Number of responses	Percentage (%)
Ain Defla	14	3.4
Ain Temouchent	4	1
Al Taref	2	0.5
Algiers (Alger)	34	8.3
Annaba	9	2.2
Batna	25	6.1
Béjaia	2	0.5
Biskra	10	2.4
Blida	39	9.5
Bordj Bou Arreridj	10	2.4
Bouira	20	4.9
Boumerdes	5	1.2
Chlef	12	2.9
Constantine	5	1.2
Djelfa	22	5.4
El Beyedh	2	0.5
El Oued	2	0.5
Ghardaia	1	0.2
Ghelizane	10	2.4
Guelma	3	0.7
Jijel	2	0.5
Khenchela	3	0.7
Laghouat	1	0.2
Mascara	5	1.2
Médéa	10	2.4
Mila	3	0.7
Mostaghanem	4	1
Msila	10	2.4
Nâama	1	0.2
Oran	21	5.1
Ouargla	6	1.5
Ouled Djellal	1	0.2
Oum El Bouaghi	15	3.7
Saida	1	0.2
Sétif	13	3.2
Sidi Belabbas	28	6.8
Skikda	3	0.7
Souk Ahras	3	0.7
Tebessa	3	0.7
Tiaret	3	0.7
Tipaza	15	3.7
Tissemsilt	1	0.2
Tizi Ouzou	6	1.5
Tlemcen	19	4.6
Touggourt	1	0.2
Total	409	100

Table S2: Crude OR (COR) of the factors associated with knowledge, attitude and practice about rabies

		Knowledge		Atitude		Practice	
		**COR (CI 95 %)**	**p value**	**COR (CI 95 %)**	**p value**	**COR (CI 95 %)**	**p value**
**Age**	18-19 yo	0.354 (0.167-0.753)	0.007	0.41 (0.195-0.862)	0.019	0.635 (0.305-1.324)	0.23
	20-29 yo	0.49 (0.234-0.91)	0.025	0.345 (0.177-0.673)	0.002	0.589 (0.305-1.137)	0.115
	>30 yo	Ref.		Ref.		Ref.	
**Sex**	Female	0.597 (0.388-0.918)	0.02	-		-	
	Male	Ref.					
**Marital status**	Married	-		1.91 (0.952-3.812)	0.069	-	
	Single			Ref.			
**Educational level**	Bachelor	0.439 (0.178-1.087)	0.076	0.966 (0.397-2.35)	0.94	0.354 (0.134-0.935)	0.036
	Masters	0.811 (0.321-2.051)	0.658	1.444 (0. 583-3.582)	0.427	0.465 (0.172-1.253)	0.123
	PG	Ref.		Ref		Ref.	
**Faculty**	MS	1.964 (1.018-3.787)	0.044	1.654 (0.866-3.160)	0.128	3.531 (1.707-7.305)	0.000
	NLS	1.649 (1.060-2.564)	0.027	0.764 (0.488-1.196)	0.24	1.569 (1.009-2.44)	0.045
	Other	Ref.		Ref.		Ref.	
**Residence**	Rural	-		1.907 (1.100-3.307)	0.022	-	
	Urban			Ref.			
**Family standard of living**	High	1.228 (0.730-2.065)	0.439	-		-	
	Low	2.3 (1.049-5.045)	0.038				
	Medium	Ref.					
**Livestock breeding**	Yes	-		1.725 (1.16-2.566)	0.007	1.295 (0.873-1.919)	0.198
	No			Ref.		Ref.	
**Dog ownership**	Yes	1.643 (0.999-2.701)	0.05	-		1.791 (1.081-2.966)	0.024
	No	Ref.				Ref.	
**Knowing one with dog bites**	Yes	1.736 (1.161-2.595)	0.007	1.421 (0.951-2.121)	0.086	1.766 (1.178-2.645)	0.006
	No	Ref.		Ref.		Ref.	
**Bitten by a dog**	Yes	-		1.702 (0.839-3.452)	0.141	1.747 (0.84-3.633)	0.135
	No			Ref.	.	Ref.	
**Knowledge score**	High	-		1.771 (1.193-2.629)	0.005	1.588 (1.074-2.347)	0.020
	Low			Ref.		Ref.	

Annex S1. Questionnaire about Knowledge, Attitude and Practice (KAP) about rabies of University Students of the endemic region in Algeria

Do you agree to participate in this survey?

Department:……………………..

Age:……………………..

Sex:……………………..

Marital status:……………………..

Educational level:……………………..

Faculty:……………………..

Residence:……………………..

Family standard of living: -Low -Medium - High

Livestock breeding: -Yes -No

Dogs ownership: -Yes -No

Do you know someone with dog bites? -Yes -No

Have you been bitten by a dog? -Yes -No

Pease answer to these questions (Rabies knowledge):

**Item**	Yes	No	I don't know
Rabies exists in Algeria			
Rabies is a deadly disease			
Infectious agents of rabies infects nerves			
All animals could be infected and transmit rabies			
Dogs are the possible source of rabies in Algeria			
Humans can be affected by rabies			
Animal bites could transmit the infectious agent to a healthy animal			
Rabies is transmitted by saliva			
Transmission from human to human by contact is possible			
Dog bites increase the risk of getting rabies			
A docile dog that suddenly turns aggressive may have rabies			
Excessive foamy salivation and the tendency to bite anything are signs of rabies in dogs			
It is against the law not to vaccinate dogs against rabies			
Age of vaccination of dogs is 3 months			
Vaccination of dogs against rabies must be repeated every year			
Registering dogs can help control rabies			

Pease answer to these questions (Rabies attitude):

Item	Yes	No	I don't know
I do not allow stray dogs to roam freely around me			
A dog that bites someone should be caught and killed			
If I am bitten by a dog, I will go to the hospital			

It is good to let dogs wander to get foods because it makes them grow quickly			
It is inhumane/bad to confine your dog(s)			
It is good not to play with unknown dogs			
Keeping dogs unvaccinated against rabies is dangerous and should be avoided			
Children should be allowed to play with dogs			

Pease answer to these questions (Rabies Practice):

<table>
<tr><th colspan="2">Item</th><th>Yes</th><th>No</th><th>I don't know</th></tr>
<tr><td colspan="2">Is it good to vaccinate your dog(s)</td><td></td><td></td><td></td></tr>
<tr><td colspan="2">Is it good to wash dog bites with soap</td><td></td><td></td><td></td></tr>
<tr><td colspan="2">It's good to have a cage for your dog(s)</td><td></td><td></td><td></td></tr>
<tr><td colspan="2">It is not a good practice to castrate/neuter dogs</td><td></td><td></td><td></td></tr>
<tr><td rowspan="5">If a person is bitten by a dog, what should be done?</td><td>Take the victim to a pharmacy for treatment</td><td></td><td></td><td></td></tr>
<tr><td>Treat with traditional medicine</td><td></td><td></td><td></td></tr>
<tr><td>Take the victim to a veterinary clinic</td><td></td><td></td><td></td></tr>
<tr><td>Take to the hospital</td><td></td><td></td><td></td></tr>
<tr><td>Nothing</td><td></td><td></td><td></td></tr>
</table>

Annex S2: STROBE Statement—checklist of items that should be included in reports of observational studies

	**Item No**	**Recommendation**
**Title and abstract**	1	(*a*) Indicate the study's design with a commonly used term in the title or the

		abstract<br>Cross-Sectional study as stated in title and abstract on the page 1
		(*b*) Provide in the abstract an informative and balanced summary of what was done and what was found<br>A summary of study is provided in the abstract on page 1
**Introduction**		
Background/rationale	2	Explain the scientific background and rationale for the investigation being reported<br>Mentioned in the introduction on page 2
Objectives	3	State specific objectives, including any prespecified hypotheses<br>Mentioned in the introduction on page 2 (Lines 86-87)
**Methods**		
Study design	4	Present key elements of study design early in the paper<br>Present in Methods section-Study design sub-heading on page 2-3
Setting	5	Describe the setting, locations, and relevant dates, including periods of recruitment, exposure, follow-up, and data collection<br>Present in Methods section-Study design sub-heading on page 3
Participants	6	(*a*) *Cohort study*—Give the eligibility criteria, and the sources and methods of selection of participants. Describe methods of follow-up<br>*Case-control study*—Give the eligibility criteria, and the sources and methods of case ascertainment and control selection. Give the rationale for the choice of cases and controls<br>*Cross-sectional study*—Give the eligibility criteria, and the sources and methods of selection of participants<br>Present in Methods section- Inclusion/Exclusion criteria sub-heading on page2-3 (Lines 99-100)
		(*b*) *Cohort study*—For matched studies, give matching criteria and number of exposed and unexposed<br>*Case-control study*—For matched studies, give matching criteria and the number of controls per case
Variables	7	Clearly define all outcomes, exposures, predictors, potential confounders, and effect modifiers. Give diagnostic criteria, if applicable<br>N/A
Data sources/ measurement	8*	For each variable of interest, give sources of data and details of methods of assessment (measurement). Describe comparability of assessment methods if there is more than one group<br>Present in Methods section-Study tool sub-heading on pages 3 (Lines 120-137)
Bias	9	Describe any efforts to address potential sources of bias<br>Present in Methods section- Inclusion/Exclusion criteria sub-heading on page 3
Study size	10	Explain how the study size was arrived at<br>Present in Methods section-Study design sub-heading on page 3 (Lines 114-118)
Quantitative variables	11	Explain how quantitative variables were handled in the analyses. If applicable, describe which groupings were chosen and why<br>Present in Methods section-Statistical analysis sub-heading on page 3 (Lines 132-137)

Statistical methods	12	(*a*) Describe all statistical methods, including those used to control for confounding<br>Present in Methods section-Statistical analysis sub-heading on page 4 (Lines 139-146).
		(*b*) Describe any methods used to examine subgroups and interactions<br>Present in Methods section-Statistical analysis sub-heading on page 4
		(*c*) Explain how missing data were addressed<br>Present in Methods section- Inclusion/Exclusion criteria sub-heading on page 4
		(*d*) *Cohort study*—If applicable, explain how loss to follow-up was addressed<br>*Case-control study*—If applicable, explain how matching of cases and controls was addressed<br>*Cross-sectional study*—If applicable, describe analytical methods taking account of sampling strategy
		(*e*) Describe any sensitivity analyses<br>No any sensitivity in analyses

Results

Participants	13*	(a) Report numbers of individuals at each stage of study—eg numbers potentially eligible, examined for eligibility, confirmed eligible, included in the study, completing follow-up, and analysed<br>Not necessary in our study
		(b) Give reasons for non-participation at each stage<br>Not necessary in our study
		(c) Consider use of a flow diagram<br>Not necessary in our study
Descriptive data	14*	(a) Give characteristics of study participants (eg demographic, clinical, social) and information on exposures and potential confounders<br>Mentioned in Results section on page 4 (Line 149-159)
		(b) Indicate number of participants with missing data for each variable of interest<br>Participants with missing data were excluded as mentioned in the methods section-Inclusion/Exclusion criteria sub-heading on page 4 (Lines 149-150)
		(c) *Cohort study*—Summarise follow-up time (eg, average and total amount)
Outcome data	15*	*Cohort study*—Report numbers of outcome events or summary measures over time
		Case-control study—Report numbers in each exposure category, or summary measures of exposure
		Cross-sectional study—Report numbers of outcome events or summary measures<br>All outcomes are mentioned in the results section on pages 4-9
Main results	16	(*a*) Give unadjusted estimates and, if applicable, confounder-adjusted estimates and their precision (eg, 95% confidence interval). Make clear which confounders were adjusted for and why they were included<br>N/A
		(*b*) Report category boundaries when continuous variables were categorized<br>N/A
		(*c*) If relevant, consider translating estimates of relative risk into absolute risk for a meaningful time period

		N/A
Other analyses	17	Report other analyses done—eg analyses of subgroups and interactions, and sensitivity analyses<br>N/A
**Discussion**		
Key results	18	Summarise key results with reference to study objectives<br>Mentioned in the discussion section on pages 9 (Lines 233-237)
Limitations	19	Discuss limitations of the study, taking into account sources of potential bias or imprecision. Discuss both direction and magnitude of any potential bias<br>Mentioned on page 11 (Lines 342-348)
Interpretation	20	Give a cautious overall interpretation of results considering objectives, limitations, multiplicity of analyses, results from similar studies, and other relevant evidence<br>As mentioned in the discussion section on pages 9-11
Generalisability	21	Discuss the generalisability (external validity) of the study results<br>Mentioned on page 11
**Other information**		
Funding	22	Give the source of funding and the role of the funders for the present study and, if applicable, for the original study on which the present article is based<br>Mentioned on page 12

*Give information separately for cases and controls in case-control studies and, if applicable, for exposed and unexposed groups in cohort and cross-sectional studies.

Note: An Explanation and Elaboration article discusses each checklist item and gives methodological background and published examples of transparent reporting. The STROBE checklist is best used in conjunction with this article (freely available on the Web sites of PLoS Medicine at http://www.plosmedicine.org/, Annals of Internal Medicine at http://www.annals.org/, and Epidemiology at http://www.epidem.com/). Information on the STROBE Initiative is available at www.strobe-statement.org.
